# Impact of coriander (*Coriandrum sativum*), garlic (*Allium sativum*), fenugreek (*Trigonella foenum-graecum*) on zootechnical performance, carcass quality, blood metabolites and nutrient digestibility in broilers chickens

**DOI:** 10.1080/01652176.2023.2300948

**Published:** 2024-02-29

**Authors:** Abdul Hafeez, Said Shahid Ali, Junaid Akhtar, Shabana Naz, Abdulwahed Fahad Alrefaei, Mohammed Fahad Albeshr, Muhammad Israr, Rifat Ullah Khan

**Affiliations:** a Department of Poultry Science, Faculty of Animal Husbandry & Veterinary Sciences, The University of Agriculture, Peshawar, Pakistan; b Department of Zoology, Government College University, Faisalabad, Pakistan; c Department of Zoology, College of Science, King Saud University, Riyadh, Saudi Arabia; d Project Management, University of Portsmouth, Portsmouth, UK; e College of Veterinary Sciences, Faculty of Animal Husbandry & Veterinary Sciences, The University of Agriculture, Peshawar, Pakistan

**Keywords:** Digestibility, broiler, phytogenics, nutrients digestability, Growth, alternative to antibiotics, blood metabolites

## Abstract

The study investigated the impact of incorporating a specific herbal blend comprising coriander, garlic, and fenugreek (CGF) at various levels on the zootechnical performance, blood metabolites and nutrient digestibility in broiler chickens. The 42-day experiment involved 360 broilers (Cobb 500), organized into four distinct treatment groups. The dietary interventions included a control group consisting of a basal diet and the same diet was supplemented with CGF at rates of 1, 2, and 3%. Broilers receiving a 1% phytogenic mixture exhibited significantly increased live weight and carcass weight. Moreover, the digestibility of crude protein and crude fat significantly improved in broilers supplemented with a 1% phytogenic mixture. On the other hand, the digestibility of calcium and phosphorus showed a notable increase in broilers fed with a 3% phytogenic mixture. Regarding serum metabolites, the 1% phytogenic mixture group displayed significantly higher levels of high density lipoprotein and triglycerides. The supplementation of the broiler diet with a herbal mixture of coriander, fenugreek, and garlic at a 1% rate resulted in improved growth performance, carcass quality, nutrient digestion, and lipid profile.

## Introduction

Antibiotics are being used as feed supplements in poultry diets to improve growth performance in farm animals (Khan, Nikousefat et al. 2012; Shehata, Attia, et al. 2022). However, alternative compounds, including probiotics, prebiotics, organic acids and natural herbs (Foroutankhah et al. 2019; Hafeez, Shah, et al. 2020; Hafeez, Sohail, et al. 2020; Alshelmani et al. 2021; Shehata, Yalçın, et al. 2022), have gained significant attention. This shift is primarily attributed to the European Union’s 2006 ban on the use of antibiotics as growth promotors in animal diets (Khan and Naz 2013; Abudabos et al. 2018; Khan et al. 2021). Feed additives are incorporated in small quantities into animal diets to improve the quality of the feed, promote animal health, and improve overall performance (Khan, Naz, Nikousefat, Tufarelli, Javdani, et al. 2012; Khan, Naz, Nikousefat, Tufarelli, and Laudadio 2012; Landy et al. 2021). These additives not only boost feed intake, nutrient utilization, and animal growth but also have an impact on stress resistance and immune function (Alzawqari et al. 2016; Saleh et al. 2021). Research suggests that herbs can positively act as effective antibiotic-free feed for broilers, leading to reduced mortality and improved growth performance without compromising consumer health (Dhama et al. 2014). Phytobiotics feed supplements, derived from herbal plants, have shown promise as antibiotic alternatives. A research indicated that these supplements may enhance feed efficiency and carcass yield (Attia et al. 2017). The important compounds found in herbs have the potential to enhance blood circulation, combat pathogens, and strengthen the immune system of broilers (Ali et al. 2019; Haq et al. 2020).

The important chemicals derived from coriander (*Coriandrum sativum*) have been identified as digestive stimulants and antioxidants (Wangensteen et al. 2004). The essential oil extracted from the seeds of coriander is predominantly composed of linalool, a compound with various pharmacological qualities, including antibacterial, insecticidal, nematicidal, antibiotic, and antimicrobial effects (Kim et al. 2008; Begnami et al. 2009; Silva et al. 2011; Khani and Rahdani 2012; Hosseinzadeh et al. 2014). Essential oils extract from coriander contains important chemicals such as a-pinene (10.5%), g-terpinene (9.0%), geranyl acetate (4.0%), camphor (3.0%), linalool (67.70%), geraniol (1.9%) and (Khani and Rahdani 2012). Supplementation of coriander has been associated with improved health parameters in broilers, as reported by Hosseinzadeh et al. (2014). Additionally, Shirazi et al. (2020) discovered that the concurrent use of coriander seeds led to better intestinal health in broilers, without impacting production performance.

The addition of garlic (*Allium sativum*) to broiler feed has shown favorable outcomes, such as enhanced growth and a reduced mortality rate, as documented by Khan, Nikousefat et al. (2012). Furthermore, the supplementation of garlic in broiler feed has been observed to promote growth in broilers (Ali et al. 2019). Garlic is known to contain essential alkaloids such as allin, allicin, sulfuric compounds and diallylsulfide, allyldisulfide, which are bioactive components with several pharmacological properties (Khan et al. 2012). A research trial concluded that garlic has the potential to serve as a growth promoter in poultry. Pagrut et al. (2018) explored the effects of incorporated garlic (0.5 kg/ton) into broiler diets, resulted in highest live weight among the treated groups, indicating a positive impact on broiler productive performance. The results imply that incorporating garlic into the diet could be an effective strategy for optimizing broiler production. Moreover, Issa and Omar (2012) noted that broilers fed with garlic demonstrated enhanced lipid profiles.

Fenugreek (*Trigonella foenumgraecum*) stands out as one of extensively cultivated plants in Asia. Notably, fenugreek has been observed to have a positive impact on the health and performance of poultry by stimulating feed intake and appetite (Wahab et al. 2019). Balasubramanian et al. (2016) suggested that the intake of fenugreek is linked with enhanced enzyme availability, improved immune system, and elevated antioxidant activity. The seeds of Fenugreek are especially rich in fat as well as various macro and micromammals (Khorshidian et al. 2019). Broilers supplemented with 1% fenugreek seeds resulted in enhancements in body weight, coupled with reductions in some important serum metabolites (Mamoun et al. 2014).

To date, there has been no study exploring the combined effects of coriander, garlic, and fenugreek (CGF) at different levels in broilers. Consequently, this study assessed the impact of CGF supplementation at 1, 2 and 3% levels on the zootechnical performance, carcass quality, blood metabolites and nutrient digestibility in broilers.

## Materials and methods

### Preparation of feed additives

The dried seeds of coriander and fenugreek along with dried bulbs of garlic, were procured from the market, milled in equal quantity, and combined at levels of 1, 2 and 3% and incorporated in the broiler feed diets.

### Experimental protocol, animals and diets

A total of 360 one-day-old male broiler chicks (Cobb 500) were acquired for the experiment. These broilers were evenly distributed into four groups, each consisting of six replicates. The broilers were raised in an open-sided system with an average temperature of 28.5 ± 1.5 °C and humidity of 63.55 ± 2.33%. Throughout the six-weeks trial period, ample ventilation and lighting were maintained. One group served as the control without any additives. Meanwhile, Groups A, B, and C were supplemented CGF at the rate of 1, 2 and 3% receptively with basic feed. The composition of dietary ingredients is specified in Table 1, and Table 2 provides the analyzed nutrient content of the herbal plants.

**Table 1.** Basal diet composition.

Name of ingredient	Starter feed (g/kg of diet)	Finisher feed (g/kg of diet)
Maize	660.0	670.0
Wheat bran	155.0	150.0
Corn oil	23.25	31.00
Corn gluten meal (32%)	91.55	60.30
Lysine	0.55	0.50
Methionine	1.10	2.00
Cotton seed cake (25%)	55.00	70.00
Lime stone	4.55	9.00
Salt	2.25	2.25
Vitamin premix^a^	3.00	3.00
Mineral premix^b^	2.55	2.55
Determined nutrient composition		
Dry matter	930.0	925.0
ME (MJI/kg)	13.50	13.6
Crude protein	245.25	215.5
Ash	85.5	85.56
Crude fibers	15.25	15.25
Ether extract	45.45	45.52

^a^ Providing per kilogram of diet:vitamin D3, 2500 IU; vitamin A, 9500 IU; vitamin K, 3 mg; vitamin E 20 IU; riboflavin, 6.6 mg; thiamine, 2.0 mg; pyridoxine, 3 mg; niacin, 30 mg; D-pantothenic acid, 100 mg; cyano-cobalamin, 20 µg; biotin, 0.1 mg; folic acid, 1.5 mg; choline, 500 mg;.

^b^ Providing per kilogram of diet: Zn, 80.0; Mn, 90 mg; Cu, 10 mg; I, 1 mg; and Se, 0.2 mg; Fe, 50 mg.

**Table 2.** Basic ingredients of coriander, garlic and fenugreek (%).

Basic ingredients	Fenugreek	Garlic	Coriander
Fat	4.13	2.46	16.9
Protein	27.6	15.6	11.9
Carbohydrate	44.5	65.0	53.0
Fiber	5.9	3.76	42.5
Moisture	3.8	5.62	8.85
Ash	3.2	5.45	4.59
Vitamin C (mg)	11.0	14.5	22.0
Vitamin A (IU)	1150	890	0.00
Pyridoxine (mg)	0.7	11.5	0.00
Niacin (mg)	6.50	4.42	2.15
Riboflavin (mg)	0.56	0.25	0.250
Thiamine (mg)	0.41	0.15	0.245
Folate (µg)	56	46	58
Nicotinic acid (mg)	1.15	13.0	0.19
Iron (mg)	32.5	5.24	15.42
Calcium (mg)	175	22.4	709
Phosphorus (mg)	295	9.55	410
Zinc (mg)	2.55	0.54	4.75
Manganese (mg)	1.32	0.16	0.145
Magnesium (mg)	192	3.99	332
Selenium (µg)	6.35	4.22	3.46

### Data collection and sampling procedure

Live weight was measured at the starter and finisher phase of the study. After slaughtering and processing broilers, carcass weight and dressing percentage were determined. Similarly, gizzard, heart and liver weight were also measured for the starter and finisher phases.

### Determination of apparent ileal digestibility

To evaluate the apparent ileal digestibility of nutrients in broilers, we introduced chromic oxide (0.5 g/kg) to serve as an inert marker. Individual cages were assigned to four broilers per treatment on 14 and 35 days of age. The experimental timeline included a four-day acclimation phase followed by three days sample collection period. Throughout the trial, excreta were collected from each replicate four times daily and stored at −20 °C. Both feed and excreta samples underwent nutrients analysis (Shuib et al. 2022). Phosphorus levels were determined using a spectrophotometer, while chromium and calcium were analyzed using an atomic absorption spectrophotometer. Phosphorus levels were determined using a spectrophotometer, while calcium were analyzed using an atomic absorption spectrophotometer.

### Analysis of blood metabolites

On day 21 of the experiment, blood samples were collected from 2 broilers per replicate to measure various serum metabolites. A chemistry analyzer, as previously mentioned, was employed for these measurements, utilizing commercial kits supplied by Zhongsheng Biochemical Company, Beijing, China. The spectrophotometer, as detailed earlier, facilitated the analysis of these blood parameters.

#### Statistical analysis

In the statistical analyses section, normality of the data was assessed using the Shapiro–Wilk test. The statistical model employed for the analysis was the General Linear Model (GLM) procedure of SAS software (2004). The experiment unit consisted of individual broilers, and the experimental design was a completely randomized design with four treatment groups. Mean differences among treatment groups were tested using one-way analysis of variance (ANOVA), and statistical significance was determined at a probability threshold of *p* < .05 using Tukey test.

## Results

### Growth performance

Data regarding live body weight, weight of carcass, giblets and dressing percentage during starter phase are presented in Table 3. Live body weight at day 21 was significantly higher for Group-C (937.0 ± 35.7) followed by Group-A (935.9 ± 88.3), B (922.8 ± 86.2) and D (822.0 ± 49.5). Carcass weight was significantly higher for Group-B (589.8 ± 62.7) followed by Group-A (570.5 ± 33.6), C (542.6 ± 25.0) and D (494.0 ± 11.5). Giblets (heart, liver and gizzard) weight was significantly not affected. Numerically higher weight for liver was noted in Group-B (19.25 ± 4.33), followed by Group-D (17.75 ± 4.80) and A (17.63 ± 6.57) and lowest for Group-C (14.63 ± 3.46). The same pattern was recorded for gizzard weight. Weight of heart was numerically higher for Group-B (5.25 ± 1.75) followed by Group**-**C (4.50 ± 2.07), D (3.75 ± 2.25) and A (3.50 ± 1.51). Data regarding dressing percentage was significantly not affected. Numerically dressing percentage was higher for Group-B (63.9 ± 4.28) followed by Group-A (61.3 ± 5.50) and D (60.3 ± 3.7) while lowest data was recorded for Group-C (57.9 ± 3.09).

**Table 3.** Effect of dietary mixture of coriander, garlic and fenugreek on live body, carcass, heart, liver, gizzard and dressing percentage at starter phase.

Groups	A	B	C	D	*p* Values
Live weight (g)	935^a^±88.3	922^a^±86.2	937^a^±35.7	822^b^±49.5	.006
Carcass weight (g)	570^a^±33.6	589^a^±62.7	542^ab^±25.0	494^b^±11.5	<.001
Gizzard weight (g)	24.1 ± 1.13	27.0 ± 4.93	23.9 ± 5.38	26.5 ± 2.27	.314
Heart weight (g)	3.50 ± 1.51	5.25 ± 1.75	4.50 ± 2.07	3.75 ± 2.25	.269
Liver weight (g)	17.6 ± 6.57	19.3 ± 4.33	14.6 ± 3.46	17.8 ± 4.80	.276
Dressing %	61.3 ± 5.50	63.9 ± 4.28	57.9 ± 3.09	60.3 ± 3.70	.059

*Notes:* Means in the same row with different superscripts are significantly different at *p* = .05. A: control; B: 1% coriander, garlic and fenugreek; C: 2% coriander, garlic and fenugreek; D: 3% coriander, garlic and fenugreek.

Data of live body weight, carcass weight, giblets weight and dressing percentage during finisher phase are presented in Table 4. Live body weight was significantly not affected. Numerically higher weight was recorded for group B (1854.3 ± 33.1) as compared to Group-A (1775 ± 130) followed by Group D (1762 ± 279) and C (1624 ± 130.9). Carcass weight was significantly higher for Group-B (1192 ± 19.5), followed by Group-A (1080 ± 42.1), D (1042 ± 144) and C (949 ± 16.2). Weight of giblets was significantly not affected. Gizzard weight was numerically highest for Group-B (39.4 ± 8.08) and D (38.6 ± 6.61) while lower for Group-A (32.0 ± 5.75) and C (31.4 ± 8.97). Heart weight was numerically higher for Group-B (10.1 ± 4.08) followed by Group-D (8.75 ± 4.77), C (8.50 ± 2.07) and A (7.50 ± 1.51). Numerically highest liver weight was observed for Group-B (40.3 ± 8.10) followed by Group-D (40.1 ± 8.18) while lower for control group (39.6 ± 6.56) and Group-C (36.3 ± 9.16). There was no significant effect on dressing percentage at finisher phase, numerically Group-B (64 ± 0.985) had higher dressing percentage compared to Group-A (61 ± 3.15) followed by Group-D (60 ± 8.26) while lowest for Group-C (59 ± 5.14).

**Table 4.** Effect of dietary mixture of coriander, garlic and fenugreek on live body, carcass, heart, liver, gizzard and dressing percentage at finisher phase.

Groups	A	B	C	D	*p* Values
Live weight (g)	1775 ± 130	1854.3 ± 33.1	1624 ± 131	1762 ± 279	.071
Carcass weight (g)	1080^b^±42.1	1192^a^±19.5	949^c^±16.2	1042^bc^±144	<.001
Gizzard weight (g)	32.0 ± 5.75	39.4 ± 8.08	31.4 ± 8.97	38.6 ± 6.61	.073
Heart weight (g)	7.50 ± 1.51	10.1 ± 4.08	8.50 ± 2.07	8.75 ± 4.77	.497
Liver weight (g)	39.6 ± 6.56	40.3 ± 8.10	36.3 ± 9.16	40.1 ± 8.18	.724
Dressing%	61.0 ± 3.15	64.0 ± 0.99	59.0 ± 5.14	60.0 ± 8.26	.189

*Notes:* Means in the same row with different superscripts are significantly different at *p* = .05. A: control; B: 1% coriander, garlic and fenugreek; C: 2% coriander, garlic and fenugreek; D: 3% coriander, garlic and fenugreek.

**Table 5.** Effect of dietary mixture of coriander, garlic and fenugreek on apparent ileal digestibility (%) of nutrients in broilers at starter phase.

	A	B	C	D	*p* Value
Dry matter	64.7 ± 2.00	66.3 ± 2.12	65.4 ± 2.75	65.3 ± 2.57	.61
Ash	45.7 ± 2.32	45.5 ± 2.31	44.9 ± 2.75	45.2 ± 1.73	.91
Crude protein	76.8 ± 3.56	79.6 ± 2.82	77.9 ± 1.88	79.3 ± 2.79	.20
Crude fat	80.3 ± 3.52	81.3 ± 4.55	81.4 ± 3.47	82.8 ± 3.72	.67
Nitrogen free extract	89.8 ± 2.65	89.7 ± 3.40	88.9 ± 1.89	89.4 ± 2.57	.92
Calcium	35.2 ± 2.43	36.1 ± 2.83	34.1 ± 2.02	35.5 ± 3.13	.50
Phosphorus	37.1 ± 2.73	37.3 ± 3.20	36.7 ± 3.38	39.0 ± 2.27	.39

*Note:* A: control; B: 1% coriander, garlic and fenugreek; C: 2% coriander, garlic and fenugreek; D: 3% coriander, garlic and fenugreek.

### Nutrient digestibility

Apparent ileal digestibility of nutrients (AID) in broiler chicken at starter phase is shown in Table 5. The supplementation of PFAs in broiler feed had no significant effect (*p*** **>** **.05) on percent apparent ileal digestibility of Dry matter, Ash, Crude Protein, Crude Fat, Nitrogen Free Extract, Calcium and Phosphorus.

Results of percent apparent ileal digestibility of nutrients in broilers during the finisher phase are shown in Table 6. Apparent ileal digestibility of ash and dry matter remained unaffected (*p*** **>** **.05), while a significantly higher (*p*** **<** **.05) AID was recorded for crude protein in Group-B (76.8±1.21) in comparison with Group-A (71.8 ± 2.09) and Group-D (73.9 ± 1.25) followed by Group-C (68.2 ± 1.29). Crude fat was recorded significantly increased (*p*** **<** **.05) in Group-B (88.3 ± 1.24) followed by Group-D (86.4 ± 1.21), Group-A (83.9 ± 1.35) and Group-C (68.2 ± 1.29). NFE showed significantly increased (*p*** **<** **.05) AID in Group-D (85.8 ± 1.36) with no differences with Group-A (84.3 ± 1.10), followed by Group-B (83.5 ± 1.00) and Group-C (81.6 ± 1.01). AID of Calcium was significantly increased (*p*** **<** **.05) in Group-D (33.9 ± 1.28) and Group-A (33.4 ± 1.31) followed by Group-B (32.8 ± 1.13), and Group-C (31.2 ± 1.11). AID of Phosphorus was recorded significantly increased (*p*** ***<*** **.05) in Group-D (33.2 ± 1.12), and Group-A (32.5 ± 1.25) in comparison with Group-C (30.8 ± 1.04) which was similar to Group-B (31.6 ± 1.15).

**Table 6.** Effect of dietary mixture of coriander, garlic and fenugreek on apparent ileal digestibility (%) of nutrients in broilers at finisher phase.

	A	B	C	D	*p* Value
Dry matter	67.5 ± 2.08	67.6 ± 1.29	67.1 ± 1.17	68.5 ± 1.52	.33
Ash	43.5 ± 1.32	43.1 ± 2.89	43.5 ± 2.59	44.1 ± 2.70	.87
Crude protein	71.8^b^±2.09	76.8^c^±1.21	68.2^a^±1.29	73.9^b^±1.25	.01
Crude fat	83.9^b^±1.35	88.3^d^±1.24	82.1^a^±1.25	86.4^c^±1.21	.01
Nitrogen free extract	84.3^bc^±1.10	83.5^b^±1.00	81.6^a^±1.01	85.8^c^±1.36	.02
Calcium	33.4^b^±1.31	32.8^ab^±1.13	31.2^a^±1.11	33.9^b^±1.28	.01
Phosphorus	32.5^b^±1.25	31.6^ab^±1.15	30.8^a^±1.04	33.2^b^±1.12	.02

*Notes:* Means presented in the same row with different superscripts are significantly different at *p* = .05; A: control; B: 1% coriander, garlic and fenugreek; C: 2% coriander, garlic and fenugreek; D: 3% coriander, garlic and fenugreek.

### Blood metabolites

Table 7 shows results for effect of PFAs on blood parameters at starter phase. Results indicated significantly decreased (*p*** **<** **.05) for triglycerides in Group-B (43.3 ± 2.47) in comparison with Group-A (47.1 ± 2.45) and Group-D (50.0 ± 2.86) with no difference in Group-C (46.8 ± 2.52). LDL, at the starter phase was significantly decreased (*p*** **<** **.05) in Group-B (37.6 ± 2.54), Group-C (40.7 ± 5.04) and Group-D (35.3 ± 3.40) in comparison with Group-A (48.3 ± 6.79). Results for high density lipoprotein (HDL) showed significantly increased (*p*** **<** **.05) for Group-B (60.2 ± 3.90) and Group-C (59.0 ± 3.38), followed by Group-D (53.9 ± 2.72) and Group-A (52.8 ± 1.68). Total Cholesterol, Total Sugar and Total Protein revealed no significant effect (*p*** **>** **.05).

**Table 7.** Effect of dietary mixture of coriander, garlic and fenugreek on blood parameters in broilers at starter phase.

Metabolites	A	B	C	D	*p* Value
Triglyceride (mg/dl)	47.1^b^±2.45	43.3^a^±2.47	46.8^ab^±2.52	50.0^b^±2.86	.01
Total cholesterol (mg/dl)	108 ± 6.35	108 ± 5.92	111 ± 4.62	114 ± 4.58	.08
LDL (mg/dl)	48.3^b^±6.79	37.6^a^±2.54	40.7^a^±5.04	35.3^a^±3.40	.01
HDL (mg/dl)	52.8^a^±1.68	60.2^b^±3.90	59.0^b^±3.38	53.9^a^±2.72	.02
Glucose (mg/dl)	12.0 ± 0.66	11.8 ± 0.68	11.7 ± 0.99	12.2 ± 0.88	.52
Total protein (g/dl)	4.19 ± 0.74	3.94 ± 0.71	4.05 ± 0.60	3.81 ± 0.58	.71

*Notes:* Means presented in the same row with different superscripts are significantly different at *p* = .05; A: control; B: 1% coriander, garlic and fenugreek; C: 2% coriander, garlic and fenugreek; D: 3% coriander, garlic and fenugreek.

## Discussion

There is an increasing interest in investigating alternatives to antibiotics in poultry diets, aiming to ensure the production of safe and high-quality food. Phytogenics have emerged as promising alternative feed additives, regulating physiological functions and promoting host organism health. In this study, live weight and carcass weight significantly decreased in group D compared to the control. Weight gain increased at the 1% level but decreased at higher doses, indicating negative impact on broilers. Existing literature provides mixed findings on the individual effects of CGF on weight gain in broilers. Their combined effects have not been previously reported, making conclusive statements challenging. Optimal production performance was observed at the 1% FGC level, while higher doses (2% and 3%) led to reduced performance, possibly due to their unfavorable smell.

In the present study, no significant difference was found in apparent ileal digestibility during the starter phase. However, group B exhibited significantly higher CP and CF during the finisher phase. Nitrogen-free extract, Ca, and P decreased significantly in group C. Interestingly, nutrients digestibility declined at higher doses of CGF mixture. Diverse phytogenic feed additives have been documented to exhibit inclusion level-dependent effects. These effects vary in magnitude and nature based on the inclusion levels applied (Paraskeuas et al. 2017). Phytogenics may enhance nutrient digestibility by improving digestion activities (Khan, Nikousefat et al. 2012). The herbal mixture enhances nutrient digestibility in broilers by promoting increased enzyme secretion, modifying intestinal microbiota, and improving overall intestinal health, as documented by Khan, Naz, Nikousefat, Tufarelli, Javdani, et al. (2012). This improvement extends to the enhancement of dry matter digestibility in the studied context.

Since this phytogenic mixture consists of many important minerals and vitamins, therefore, these may have played important role.

In the present study, TG decreased significantly in broiler chickens supplemented with 2% and 3% of the phytogenic mixture. However, LDL increased in all the treatment groups. Conversely, HDL concentration decreased in broilers supplemented with 3% of the phytogenic mixture. Using each of these herbs individually has been reported to enhance growth and blood metabolites in broilers (Abdalla et al. 2011; Saleh et al. 2014). The enhanced blood profile is attributed to the antioxidant activities of the herbs, which reduce lipid peroxidation (Khan, Nikousefat et al. 2012). Additionally, these herbs may potentially inhibit the Acetyl CoA synthetase enzyme, a rate-limiting enzyme in cholesterol synthesis (Khan, Naz, Nikousefat, Tufarelli, and Laudadio 2012).

## Conclusions

Supplementing the broiler diet with a 1% herbal blend comprising coriander, fenugreek, and garlic led to enhanced growth performance, carcass quality, nutrient digestion, and lipid profile.

## Data Availability

These data are available in student thesis.
